# Patterns of Cervical Lymph Node Metastasis in Locally Advanced Supraglottic Squamous Cell Carcinoma: Implications for Neck CTV Delineation

**DOI:** 10.3389/fonc.2020.01596

**Published:** 2020-08-27

**Authors:** Yi Xu, Ye Zhang, Zhengang Xu, Shaoyan Liu, Guozhen Xu, Li Gao, Jingwei Luo, Xiaodong Huang, Kai Wang, Yuan Qu, Shiping Zhang, Qingfeng Liu, Runye Wu, Xuesong Chen, Junlin Yi

**Affiliations:** ^1^Department of Radiation Oncology, National Cancer Center/National Clinical Research Center for Cancer/Cancer Hospital, Chinese Academy of Medical Sciences and Peking Union Medical College, Beijing, China; ^2^Department of Head and Neck Surgery, National Cancer Center/National Clinical Research Center for Cancer/Cancer Hospital, Chinese Academy of Medical Sciences and Peking Union Medical College, Beijing, China

**Keywords:** locally advanced, supraglottic squamous cell carcinoma, lymph node metastasis, clinical target volume, delineation

## Abstract

**Objective:**

To investigate the prevalence and distribution of cervical lymph node metastasis (LNM) in locally advanced supraglottic squamous cell carcinoma (LASCC) and guide the delineation of clinical lymph node target volumes.

**Materials and Methods:**

We reviewed patients defined as LASCC from January 2000 to December 2017 in our hospital. The primary tumor was operated on using partial or total laryngectomy, and all patients underwent bilateral neck dissection (levels II–IV at least). Univariate and multivariate logistic regressions were used to find risk factors associated with LNM.

**Results:**

A total of 206 patients were enrolled. In the whole group, the rate of ipsilateral metastasis (IM) was 60.9% (67 patients), whereas contralateral metastasis was 25.5% (28 patients). Only positive ipsilateral lymph nodes contributed to contralateral metastasis (*p* = 0.001). Seventy-six cases were diagnosed with clinical positive lymph nodes (cN^+^). IM of primary lesions mainly located within the unilateral sites (*n* = 49 patients) was detected in levels II, III, and IV with lymph node metastasis ratios of 73.5% (36 patients), 63.3% (31 patients), and 20.4% (10 patients), respectively, and contralateral metastasis of 36.7% (18 patients), 16.3% (8 patients), and 6.1% (3 patients), respectively. Involvement of level II or III was associated with metastasis of level IV. No one developed contralateral level IV involvement without metastasis of contralateral levels II and III. A total of 130 cases had clinically negative neck lymph nodes (cN0). The prevalence of occult metastasis (OM) was 35.4%. Among 62 patients with unilateral lesions, the rates of OM to ipsilateral neck levels II, III, and IV were 21, 11.1, and 1.6%, respectively, whereas contralateral neck levels were 6.3, 4.8, and 0%, respectively. In terms of the risk factors, histopathological differentiation was related to OM (*p* = 0.003). Two of 25 people were with level VIb metastasis, and both of them were with subglottic involvement.

**Conclusion:**

Neck levels II to IV are most frequently involved and should be included in clinical target volume (CTV) in cN^+^ patients. Contralateral IV may be omitted when contralateral levels II and III are negative. In cN0 patients, ipsilateral levels II and III are suggested to be included in the CTV, whereas whether contralateral levels II and III should be included needs further research.

## Introduction

Laryngeal cancer still ranks high in incidence rate among the upper aerodigestive tract (1), and 85–95% of these cancers are squamous cell carcinomas (2). Locally advanced laryngeal cancers are inclined to metastasize (3–6), and supraglottic cancers have the highest prevalence of regional metastases among laryngeal cancers (4). Concurrent chemoradiotherapy is offered as an alternative treatment option to improve quality of life for patients by preserving the larynx (7, 8). For most patients with T3 or T4 disease without tumor invasion through cartilage into soft tissues, concurrent chemoradiotherapy is an appropriate approach (9). However, the detailed lymph node regions that should be included in clinical target volumes (CTV) are still controversial (10, 11), especially level IV and contralateral lymph node levels in patients with cN0. Guidelines for radiotherapy in Danish Head and Neck Cancer Group (DAHANCA) suggest that bilateral levels II and III should be included in CTV in cN0 patients of locally advanced supraglottic squamous cell carcinoma (LASCC) (12), whereas in international consensus bilateral level IV is also advised (10). Additionally, whether contralateral level IV should be defined as irradiation region among patients with N + stage has not reached an agreement. Furthermore, most guidelines do not take risk factors of lymph node metastasis (LNM), such as T stage, histopathological differentiation, tumor subsite, and so on, into consideration for selection and delineation of lymphatic CTVs. We designed this study to investigate the patterns and risk factors of cervical LNM in LASCC to help guide individualized delineation of neck CTV for radical radiotherapy.

## Materials and Methods

### Patients Selection and Evaluation

Patients treated between January 1, 2000, and December 31, 2017, were selected for retrospective analysis. Eligibility criteria included the following: (1) supraglottic cancer with the histologic type of squamous cell carcinoma; (2) late T stage identified by pathological evaluation (pT3 or pT4), in accordance with the seventh American Joint Committee on Cancer tumor–node–metastasis staging system; (3) all patients must undergo computed tomography (CT) scan, ultrasound detection and clinical palpation of the neck, laryngoscope, chest CT, abdominal ultrasonography, and some obtained upper and middle abdomen CT (22.3%) or whole-body nuclear medicine bone scanning (81.5%) before surgery; magnetic resonance imaging of the neck (10.2%) and positron emission tomography–CT (PET/CT) scan (0%) were not necessary; (4) patients were primarily treated by surgery with partial or total laryngectomy for primary tumor and bilateral neck dissection (levels II–IV at least) with complete data in the standard manner of neck node levels. The exclusion criteria were as follows: (1) patients with distant metastases or synchronous tumors at first diagnosis; (2) previously received chemotherapy/radiotherapy or who had been treated elsewhere; (3) past malignancies history (except for stage I non-melanoma skin cancer or cervical carcinoma *in situ*). This study involving human participants was reviewed and approved by the Ethics Committee of the National Cancer Center/Cancer Hospital, Chinese Academy of Medical Sciences.

The patients with cN0 were diagnosed by a combination of palpation, CT scan, and/or ultrasound detection before neck dissection. The evaluation criteria of contrast-enhanced CT scan were as follows: lesser diameter <10 mm, absence of central necrosis, and absence of contrast enhancement of lymph node capsule. Clinical examination revealed that lymph nodes were <2 cm in diameter and soft (13). Meanwhile, ultrasound imaging reported no positive lymph nodes.

**FIGURE 1:**
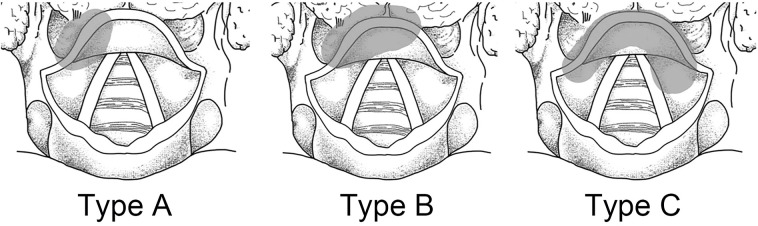
Location of primary lesions.

According to the location of primary lesions, the patients were defined as the following types: type A, unilateral without midline involvement; type B, unilateral with crossing the midline; type C, central. Contralateral metastasis (CM) was analyzed only in types A and B. LNM of the left and right neck side was described in type C (Figure 1) (14).

### Statistical Analysis

The LNM ratios (LMRs) were calculated for different levels in three groups. Neck levels with positive lymph nodes or containing a risk of occult metastases of 10–15% or more were suggested to be included in CTV (15). All parameters likely to influence LNM, including sex, age, smoking, tumor location, histological differentiation, primary subsite, T stage, and ipsilateral metastasis (IM), were analyzed by using univariate analysis. Multivariate logistic regression contained all statistically significant variables in the initial univariate analysis (*P* < 0.2). All statistical analyses were performed using SPSS 23.0 software package (IBM Inc., New York, NY, United States). A probability value of <0.05 was considered statistically significant.

## Results

### Patient Characteristics

A total of 206 patients who met the criteria were analyzed. There were 185 men and 21 women, with ages ranging from 24 to 84 years (median age, 60 years). A total of 110 (53.4%) patients had unilateral lesions, and 53.4% (130/206) were clinically staged as N0. The basic clinical characteristics of the patients and the distribution of patients by T stage, N stage, primary subsites, differentiation, and tumor location are shown in Table 1.

**TABLE 1:** Patient characteristics.

**Characteristics**	**No**	**Constituent ratio (%)**
**Sex**		
Male	185	89.8
Female	21	10.2
**Age (y)**		
≤60	113	54.9
>60	93	45.1
**Smoking**		
Yes	192	93.2
No	14	6.8
**T stage**		
T3	174	84.5
T4	32	15.5
**pN stage**		
pNO	83	40.3
pNl	42	20.4
pN2	80	38.8
pN3	1	0.5
**Primary subsite**		
Epiglottis	167	57.4
Aryepiglottic folds	10	4.9
False vocal cord	26	12.6
Ventricles	3	1.5
**Differentiation**		
Well	24	11.7
Moderately	135	65.5
Poorly	47	22.8
**Tumor location**		
Unilateral (type A and B)	110	53.4
Centrally (type C)	96	46.6

*Abbreviation: cN stage, clinical node stage; pN stage, pathologicaI node stage.*

### Distribution of LNM According to Different Clinical LN Status

#### Whole Group

A total of 10,631 lymph nodes were resected, and the average number of dissected lymph nodes was 50.5 per person (range, 5–129). Only nine patients had dissected lymph node counts of fewer than 18. The median number of positive lymph nodes was 2 per person (range, 1–49). LNM was found in 123 of the 206 (59.7%) patients. The LMRs of levels II, III, and IV were 44.2% (91), 37.4% (77), and 8.7% (18), respectively. One hundred ten cases were with unilateral tumors (types A and B). In type A patients (54), the prevalence rates of IM in levels II to IV were 37% (20), 31% (17), and 5.6% (3), respectively, and those of CM were 14.8% (8), 7.4% (4), and 1.9% (1), respectively. While in type B patients (56), the rates of IM in levels II to IV were 51.8% (29), 37.5% (21), and 14.3% (8), respectively, and those of CM were 25% (14), 10.7% (6), and 3.6% (2), respectively.

#### Clinical Positive Lymph Node (cN^+^)

**FIGURE 2:**
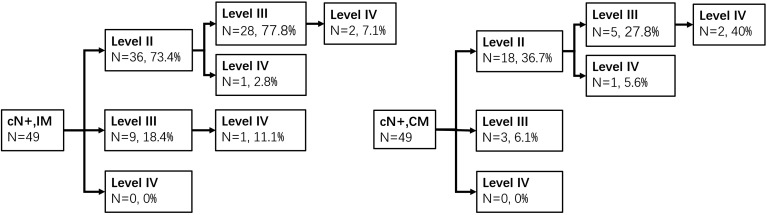
Ipsilateral and contralateral lymph drainage to each neck level in cN^+^ patients of types A and B groups.

Seventy-six cases were with cN^+^, and all of them conformed with pathologic LNM. IM of unilateral lesions (type A or B, *n* = 49) was detected in levels II, III, and IV with LMRs of 73.5% (36), 63.3% (31), and 20.4% (10), respectively, and CM of 36.7% (18), 16.3% (8), and 6.1% (3), respectively. The pathways of IM and CM were shown as follows (Figure 2). In type C (*n* = 27) group, the LMRs at left neck levels II, III, and IV were 51.8% (14), 40.7% (11), and 0% (0), respectively, and 48.1% (13), 40.7% (11), and 14.8% (4) for the right neck. There was no one who developed isolated level IV metastasis.

#### Clinically Negative Lymph Node (cN0)

**FIGURE 3:**
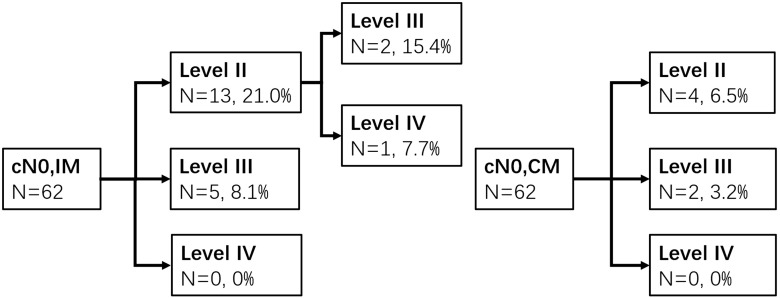
Ipsilateral and contralateral lymph drainage to each neck level in cN0 patients of types A and B groups.

The prevalence of occult metastasis (OM) was 35.4% in cN0 patients (*n* = 130). Metastasis was detected in levels II, III, and IV with LMRs of 23.8% (31), 22.3% (29), and 2.3% (3). Sixty-two patients were type A or B. The rates of OM to ipsilateral neck levels II, III, and IV were 21% (13), 11.1% (7), and 1.6% (1), respectively, whereas the contralateral neck levels were 6.3% (4), 4.8% (3), and 0% (0), respectively. The pathways of IM and CM were shown as follows (Figure 3). In type C (*n* = 68), the LMRs at left neck levels II, III, and IV were 16.2% (11), 20.6% (14), and 2.9% (2), respectively, and 16.2% (11), 16.2% (11), and 1.5% (1) for the right neck.

Twenty-five patients had level VIb dissection. The median number of dissected lymph nodes was 2 (range, 0–7). In most patients (80%, *n* = 20), lymph node counts were no more than 2. LNMs of level VIb were found in 2 (8%) patients, and both of them were with subglottic region involved.

### Factors Related With LNM

In the whole group, unilateral tumors crossing the midline (*p* = 0.041) and IM (*p* = 0.001) were associated with CM by univariate analysis. The occurrence of CM was higher in unilateral lesions (type A or B) with IM (26 of 67, 38.8%) than without (2 of 43, 4.7%). By multivariate analysis, only IM (*p* = 0.001, HR = 12.169, 95% confidence interval = 2.689–55.075) showed a significant association with CM (Table 2).

**TABLE 2:** Predictive factors of CM and ipsilateral level IV metastases in LASCC.

**Factor**	**Groups**	**CM, *P*-value**		**Level IV, *P*-value**	
		**Univariate**	**Multivariate**	**Univariate**	**Multivariate**
Primary subsite	Epiglottis Aryepiglottic- folds False vocal cord Ventricles	0.543	–	0.202	–
T stage	T3 T4	0.843	–	0.264	–
Differentiation	Well Moderately Poorly	0.547	–	0.381	–
TL	Type A Type B	0.041	0.106 (OR 2.227,0.844–5.873)	0.140	0.184 (OR 2.833,0.610–13.156)
IM	NO N+	0.001	0.001 (OR 12.169,2.689–55.075)	–	–
Level II	Positive Negative	–	–	0.011	0.032 (OR 10.508,1.225–90.115)
Level III	Positive Negative	–	–	0.003	0.013 (OR 8.257,1.558 –43.755)

*Abbreviation: CM, contralateral metastasis; TL, tumor location; IM, ipsilateral metastasis.*

Sixty (78.9%) and 48 (63.2%) patients had level II or III metastasis in cN^+^ group, so we focused on the risk factors associated with LNM in level IV. Observed by multivariate analysis, the presence of LNM in ipsilateral level IV was related to involvement of ipsilateral level II (20.4 vs. 1.6%) or III (23.7 vs. 2.8%) (Table 2).

In cN0 patients, pathological differentiation (*p* = 0.003, HR = 2.656, 95% confidence interval = 1.382–5.105) was closely associated with OM. In terms of LNM, moderately and poorly differentiated lesions were with higher incidence to levels II to IV (moderately 20, 26.7, 2.2; poorly 50, 50, 7.1%, respectively) than well-differentiated lesions (11.1, 11.1, 0%). Smoking status and primary subsite seemed to be risk factors for CM by univariate analysis. However, in the multivariate analysis, there was no significant association (Table 3).

**TABLE 3:** Predictive factors of LNM and CM in LASCC of cNO.

**Factor**	**Groups**	**LNM, *P*-value**	**CM, *P*-value**
	
		**Multivariate**	**Multivariate**
Sex	Male Female	0.756	0.553
Age (y)	≤60 >60	0.871	0.899
Smoking	Yes No	0.924	0.119
Primary subsite	Epiglottis Aryepiglottic-folds False vocal cord Ventricles	0.856	0.770
T stage	T3 T4	0.489	0.794
Differentiation	Well Moderately Poorly	0.003	0.241
Tumor location	Type A Type B	–	0.717

*Abbreviation: LNM, lymph node metastasis; CM, contralateral metastasis; LASCC, locally advanced supraglottic squamous cell carcinoma.*

## Discussion

The present study showed that the prevalence of LNM was high in level II (23.8–78.9%) and level III (22.3–63.2%), whereas it was relatively low in level IV (2.3–19.7%) in different subgroups. Ipsilateral LMRs of levels II, III, and IV were higher than contralateral (73.5 vs. 36.7%, 63.3 vs. 16.3%, and 20.4 vs. 6.1%) in cN^+^ patients; 35.4% of cN0 patients had OM, and 23.8% of them were with LNM in level II, 22.3% in level III, and 2.3% in level IV. In cN0 patients of type A, only 4 (12.9%) patients had CM, and the prevalence rates of contralateral levels II, III, and IV were 6.5% (2 of 31), 6.5% (2 of 31), and 0%, respectively. There was no one of type B with contralateral level IV metastasis.

In general, supraglottic cancer appears to have a higher rate of CM than glottic and subglottic cancer (16). Marks et al. (17) described CM in 7–26% of supraglottic cancers. Some retrospective studies reported that tumor crossing the midline and positive ipsilateral lymph node were important determinants of contralateral involvement. In the present study, 26 of 67 (38.8%) patients with ipsilateral pN^+^ had positive lymph nodes in the contralateral neck, compared to 2 of 43 (4.7%) patients with ipsilateral pN0. As numerous studies have identified, ipsilateral pathological staging (pN) is a reliable risk factor for CM (17, 18). When there were histologically confirmed metastases on the ipsilateral side, CM occurred in up to 48% of the cases in stages III and IV (18). But there is no consensus about whether the degree of midline crossing affects contralateral lymphatic spread. Some investigators found that midline involvement was a risk factor for CM (4, 19). However, these studies included not only supraglottic cancer but also glottic cancer, which accounted for the majority. Other studies proposed contradicting results (5, 18, 20). Yılmaz et al. divided the degree of midline crossing at the epiglottis into three groups, which was measured on a laryngectomy specimen with a ruler and expressed as “no or zero,” “<5 mm,” or “≥5 mm.” There was no statistically significant difference between the rates of CM in these groups. Amar et al. arrived at the same conclusion (18). As shown in other studies, crossing the midline was not a reliable risk indicator of CM in our study.

As reported in our study, where 35.4% of cN0 patients had positive lymph nodes, LASCC has a known tendency to develop OM. The rate of OM tended to increase with the progression of T staging and pathology differentiation (21, 22). In other series, occult LMRs for T3 and T4 lesions were 22.7–32.5% and 31.2–35.7%, respectively, which were higher than T1 and T2 lesions (21). In our observation, the occult LMR of T4 stage (41.2%) was relatively higher than T3 stage (34.5%), but the difference was not statistically significant (*p* = 0.489). Meanwhile, we found that the increase in grade of histopathological differentiation developed the increasing prevalence of LNM, as previous studies identified (22, 23). This result was in accord with the biological behavior of malignancies. Poorly differentiated cells possess a greater capacity to invade lymphatic vascular spaces, which enable tumor cells to enter the bloodstream and lymphatic channels and disseminate widely in the body (24), indicating that poor differentiation should be considered when we design CTV delineation.

In cN^+^ patients, levels II and III were usually first involved in either IM or CM, which is similar to the results of Tomik et al. (3), regardless of tumor subsites (25). This is mainly due to the lymphatic drainage that extends transversely and drains into nodes located at the junction of levels II and III in the supraglottic larynx (26). In type C group, the LMRs of bilateral level II or III were both more than 40%. Besides, the rates of IM in level II or III were even more than 60% in types A and B groups, and patients with unilateral LNM were likely to develop CM. Therefore, bilateral levels II and III should be included in CTV in cN^+^ patients. Many patients also had ipsilateral level IV involved (20.4%), whereas the LMR of contralateral level IV was low (6.1%). The present study showed that level II or III involvement was related to level IV metastasis by multivariate analysis. As numerous studies identified, “skip metastasis” (lymph node involvement bypasses a lymph node level and involves the next but one level) was rare, which meant that level IV was mostly affected in conjunction with the upper level (21, 27). Furthermore, no one had contralateral level IV metastasis when contralateral levels II and III were not involved in our study. Thus, bilateral level IV is suggested to be included in CTV, unless contralateral levels II and III have no LNM. In this situation, we consider omitting contralateral level IV irradiation.

The overall metastatic rate of cN0 patients was greater than 20% in pathologic examination (21, 22, 24), while the LMRs of contralateral levels II to III were relatively low in types A and B groups in our study. IM was detected in levels II and III with LMRs of 21 and 11.1%, respectively, while CM was detected in 6.3 and 4.8%, respectively. We recommend that ipsilateral levels II to III are included in CTV, whereas contralateral levels II to III may even not be irradiated in some selective cases. Recently, a clinical trial comparing bilateral neck dissection with ipsilateral neck dissection of cN0 supraglottic laryngeal cancer is ongoing in our hospital, which would provide data for shedding light on whether to irradiate the contralateral neck in the future (NCT03392220).

Whether bilateral level IV should be included in CTV is still controversial in different guidelines (10, 12, 13). As cervical extent of LNM follows a pattern, head and neck surgeons recommend omitting bilateral level IV resection for their low LMRs in previous studies (28, 29). Similarly, level IV metastasis was rare in cN0 patients of three types in our study, and there was no one with contralateral level IV metastasis in types A and B. The results of our study are consistent with DAHANCA guidelines in which bilateral level IV was excluded from CTV.

As previous studies showed, subglottic extension contributed to metastases of level VIb (27, 30). Garas et al. reported 26.6% patients with subglottic squamous cell carcinoma had spread to paratracheal nodes. Gorphe et al. (31) showed that level VIb involvement was associated with pathologic subglottic extension, lysis of the cricoid cartilage, and tracheal extension. In our cases, not all patients with subglottic involvement underwent level VIb dissection, due to different cognizance of the criterion about level VIb dissection by surgeons. But both patients who had positive lymph nodes in level VIb were with subglottic involvement. Level VIb should be included in CTV when high risk factors above exist.

Our study had several limitations. First, the lymph nodal stations were assigned according to surgical definition, which was partially different from the guideline based on CT image. Besides, cN0 was diagnosed without using PET/CT, as it was too expensive with similarity to conventional imaging for the diagnosis of neck metastasis (32). Second, we chose the patients with bilateral neck dissection who might have more of a tendency for LNM as judged by a surgeon than those with high-selective neck dissection. Third, the T3 and T4 stages were identified by pathological evaluation, which did not match the clinical T stage completely. Furthermore, as a retrospective design characterized by a long-time span, selection biases and imbalances existed in inherent variables. We failed to evaluate LNM state in each level, and did not find risk factors associated to CM in cN0 patients, which may be due to the small sample (only 6 patients with CM). Last, more subgroup analyses need to be validated in the future.

## Conclusion

The rates of neck LNMs are high in patients with LASCC, especially within levels II and III. In cN^+^ patients, bilateral levels II to IV with high risk of metastasis should be included in CTV. However, contralateral level IV may be excluded from CTV, when no positive lymph node occurs in contralateral levels II and III. In cN0 patients, ipsilateral levels II to III are suggested to be irradiated. Contralateral levels II to III may be omitted from radiation therapy fields because of their low risk of metastasis in our study.

## Data Availability Statement

All datasets generated for this study are included in the article/supplementary material.

## Ethics Statement

The studies involving human participants were reviewed and approved by Ethics Committee of National Cancer Center/Cancer Hospital, Chinese Academy of Medical Sciences and Peking Union Medical College. The patients/participants provided their written informed consent to participate in this study.

## Author Contributions

YX: writing the protocol, reviewing all the case record forms for eligibility and protocol violation, recruiting patients, and writing the manuscript. YZ and JY: study design and project administration. ZX, SL, GX, and LG: resources and original draft. XH and JL: response for patients accrual and data collection. KW, YQ, XC, QL, SZ, and RW: response for the patients data collection and data management. All authors contributed to the article and approved the submitted version.

## Conflict of Interest

The authors declare that the research was conducted in the absence of any commercial or financial relationships that could be construed as a potential conflict of interest.

## References

[1] Chen W Zheng R Baade PD Zhang S Zeng H Bray F Cancer statistics in China, 2015. *CA: A Cancer Journal for Clinicians* (2016) 66:115–32. 10.3322/caac.21338 26808342

[2] American Cancer Society. *Journalist’s Resource: American Cancer Society: Cancer Facts and Figures 2012.* New York, NY: American Cancer Society (2012).

[3] Tomik J Składzień J Modrzejewski M. Evaluation of cervical lymph node metastasis of 1400 patients with cancer of the larynx. *Auris Nasus Larynx.* (2001) 28:233–40. 11489367 10.1016/s0385-8146(00)00116-4

[4] Redaelli De Zinis LO Nicolai P Tomenzoli D Ghizzardi D Trimarchi M Cappiello J The distribution of lymph node metastases in supraglottic squamous cell carcinoma: therapeutic implications. *Head Neck.* (2002) 24:913–20. 10.1002/hed.10152 12369069

[5] Mutlu V Ucuncu H Altas E Aktan B. The relationship between the localization, size, stage and histopathology of the primary laryngeal tumor with neck metastasis. *Eur J Med.* (2014) 46:1–7. 10.5152/eajm.2014.01 25610286 PMC4261447

[6] Waldfahrer F Hauptmann B Iro H. Lymph node metastasis of glottic laryngeal carcinoma. *Laryngorhinootologie.* (2005) 84:96–100. 15712044 10.1055/s-2004-826075

[7] Strojan P Haigentz M Bradford CR Wolf GT Hartl DM Langendijk JA Chemoradiotherapy vs. total laryngectomy for primary treatment of advanced laryngeal squamous cell carcinoma. *Oral Oncol.* (2013) 49:283–6. 23219843 10.1016/j.oraloncology.2012.11.002

[8] Jencke F Knecht R. State of the art in the treatment of laryngeal cancer. *Anticancer Res.* (2013) 33:4701–10. 24222104

[9] Pfister DG Laurie SA Weinstein GS Mendenhall WM Adelstein DJ Ang KK American society of clinical oncology clinical practice guideline for the use of larynx-preservation strategies in the treatment of laryngeal cancer. *J Clin Oncol Off J Am Soc Clin Oncol.* (2006) 24:3693. 10.1200/jco.2006.07.4559 16832122

[10] Biau J Lapeyre M Troussier I Budach W Giralt J Grau C Selection of lymph node target volumes for definitive head and neck radiation therapy?: a 2019 Update. *Radiother Oncol.* (2019) 134:1–9. 10.1016/j.radonc.2019.01.018 31005201

[11] Grégoire V Ang K Budach W Grau C Hamoir M Langendijk JA Delineation of the neck node levels for head and neck tumors: A 2013 update. DAHANCA, EORTC, HKNPCSG, NCIC CTG, NCRI, RTOG, TROG consensus guidelines. *Radiother Oncol.* (2014) 110:172–81. 10.1016/j.radonc.2013.10.010 24183870

[12] Dahanca/Dshho publications. *Nationale Retningslinier for Pharynx- og Larynxcancer.* (2014). Available online at: https://www.dahanca.oncology.dk/page1.html#header1-u (accessed January 16, 2019).

[13] Kowalski LP Bagietto R Lara JR Santos RL Silva JF Jr. Prognostic significance of the distribution of neck node metastasis from oral carcinoma. *Head Neck.* (2015) 22:207–14. 10.1002/(sici)1097-0347(200005)22:33.0.co;2-9 10748442

[14] Zhang Y Xu S Liu W Wang X Wang K Liu S Rational choice of neck dissection in clinically N0 patients with supraglottic cancer. *Head Neck.* (2020) 42:365–73. 10.1002/hed.26014 31724760

[15] Chao KSC Ii FJW Ozyigit G Tran BN Dempsey JF. Determination and delineation of nodal target volumes for head-and-neck cancer based on patterns of failure in patients receiving definitive and postoperative IMRT. *Int J Radiat Oncol Biol Phys.* (2002) 53:1174–84. 10.1016/S0360-3016(02)02881-X 12128118

[16] Öztürkcan S Katilmiķ H Özdemir I Tuna B Güvenç IA Dündar R. Occult contralateral nodal metastases in supraglottic laryngeal cancer crossing the midline. *Eur Arch Oto Rhino Laryngol.* (2009) 266:117–20. 10.1007/s00405-008-0721-x 18542980

[17] Marks JE Devineni VRHJ. The risk of contralateral lymphatic metastases for cancers of the larynx and pharynx. *Am J Otolaryngol.* (1992) 13:34 10.1016/0196-0709(92)90095-B 1585983

[18] Amar A Chedid HM Franzi SA Rapoport A. Neck dissection in squamous cell carcinoma of the larynx. Indication of elective contralateral neck dissection. *Braz J Otorhinolaryngol.* (2012) 78:7–10. 10.1590/S1808-86942012000200002 PMC9443838 22499363

[19] Ferlito A Silver CE Rinaldo A. Selective neck dissection (IIA, III): a rational replacement for complete functional neck dissection in patients with N0 supraglottic and glottic squamous carcinoma. *Laryngoscope.* (2008) 118:676–9. 10.1097/MLG.0b013e31815f6f25 18182969

[20] Yılmaz T Süslü N Atay G Günaydın RÖ Bajin MD Özer S The effect of midline crossing of lateral supraglottic cancer on contralateral cervical lymph node metastasis. *Acta Oto Laryngologica.* (2015) 135:484. 10.3109/00016489.2014.986759 25677524

[21] Ma H Lian M Feng L Li P Hou L Chen X Factors contributing to lymph node occult metastasis in supraglottic laryngeal carcinoma cT2-T4 N0M0 and metastasis predictive equation. *Chin J Cancer Res.* (2014) 26:685–91. 10.3978/j.issn.1000-9604.2014.12.06 25561766 PMC4279208

[22] Esposito EDAVE Motta S Cassiano B Motta G. Occult lymph node metastases in supraglottic cancers of the larynx. *Otolaryngol Head Neck Surgery.* (2001) 124:253–7. 10.1067/mhn.2001.113146 11240985

[23] Ozdek A Sarac S Akyol MU Unal OF Sungur A. Histopathological predictors of occult lymph node metastases in supraglottic squamous cell carcinomas. *Eur Arch Oto Rhino Laryngol.* (2000) 257:389. 10.1007/s004050000231 11052252

[24] Liu YH Du ZW. Management of clinically negative nodes (N0) in supraglottic laryngeal carcinoma: a systematic review. *Genet Mol Res.* (2016) 15:179. 10.4238/gmr15048179 27813558

[25] Rivière D Mancini J Santini L Giovanni A Dessi P Fakhry N. Lymph-node metastasis following total laryngectomy and total pharyngolaryngectomy for laryngeal and hypopharyngeal squamous cell carcinoma: frequency, distribution and risk factors. *Eur Ann Otorhinolaryngol Head Neck Dis.* (2018) 135:163–6. 10.1016/j.anorl.2017.11.008 29277379

[26] Mukherji SK Armao D Joshi VM. Cervical nodal metastases in squamous cell carcinoma of the head and neck: what to expect. *Head Neck.* (2001) 23:995. 10.1002/hed.1144 11754505

[27] Garas J McGuirt WF. Squamous cell carcinoma of the subglottis. *Am J Otolaryngol.* (2006) 27:1–4. 10.1016/j.amjoto.2005.05.004 16360814

[28] Lim YC Choi EC Lee JS Koo BS Song MH Shin HA. Is dissection of level IV absolutely necessary in elective lateral neck dissection for clinically N0 laryngeal carcinoma? *Oral Oncol.* (2006) 42:101–6. 10.1016/j.oraloncology.2005.06.019 16143563

[29] Khafif A Fliss DM Gil Z Medina JE. Routine inclusion of level IV in neck dissection for squamous cell carcinoma of the larynx: Is it justified? *Head Neck.* (2004) 26:309–12. 10.1002/hed.10390 15054733

[30] Remco de B Leemans CR Silver CE Robbins KT Rodrigo JP Rinaldo A Paratracheal lymph node dissection in cancer of the larynx, hypopharynx, and cervical esophagus: the need for guidelines. *Head Neck.* (2011) 33:912–6. 10.1002/hed.21472 20652978

[31] Gorphe P Matias M Moya-Plana A Tabarino F Blanchard P Tao Y Results and survival of locally advanced AJCC 7th edition T4a laryngeal squamous cell carcinoma treated with primary total laryngectomy and postoperative radiotherapy. *Ann Surg Oncol.* (2016) 23:2596–601. 10.1245/s10434-016-5217-0 27034080

[32] Cacicedo J Navarro A Del Hoyo O Gomez-Iturriaga A Alongi F Medina JA Role of [18F] fluorodeoxyglucose PET/CT in head and neck oncology: the point of view of the radiation oncologist. *Br J Radiol.* (2016) 89:20160217. 10.1259/bjr.20160217 27416996 PMC5124833

